# Mutagenesis of mNeptune Red-Shifts Emission Spectrum to 681-685 nm

**DOI:** 10.1371/journal.pone.0148749

**Published:** 2016-04-27

**Authors:** ZhaoYang Li, ZhiPing Zhang, LiJun Bi, ZongQiang Cui, JiaoYu Deng, DianBing Wang, Xian-En Zhang

**Affiliations:** 1 National Laboratory of Biomacromolecules, Institute of Biophysics, Chinese Academy of Sciences, Beijing 10010, China; 2 State Key Laboratory of Virology, Wuhan Institute of Virology, Chinese Academy of Sciences, Wuhan 430071, China; AntiCancer Inc., UNITED STATES

## Abstract

GFP-like fluorescent proteins with diverse emission wavelengths have been developed through mutagenesis, offering many possible choices in cellular and tissue imaging, such as multi-targets imaging, deep tissue imaging that require longer emission wavelength. Here, we utilized a combined approach of random mutation and structure-based rational design to develop new NIR fluorescent proteins on the basis of a far-red fluorescent protein, mNeptune (Ex/Em: 600/650 nm). We created a number of new monomeric NIR fluorescent proteins with the emission range of 681–685 nm, which exhibit the largest Stocks shifts (77–80 nm) compared to other fluorescent proteins. Among them, mNeptune681 and mNeptune684 exhibit more than 30 nm redshift in emission relative to mNeptune, owing to the major role of the extensive hydrogen-bond network around the chromophore and contributions of individual mutations to the observed redshift. Furthermore, the two variants still maintain monomeric state in solution, which is a trait crucial for their use as protein tags. In conclusion, our results suggest that there is untapped potential for developing fluorescent proteins with desired properties.

## Introduction

Fluorescent proteins (FPs) are widely used as protein tags for cellular and in vivo molecular targeting as well as tracking. In mammals, tissues are most transparent to light in the near-infrared (NIR) optical window (650 nm–900 nm), largely due to minimal absorption at this range of water, melanin, and hemoglobin [1,2].

In recent years, a number of NIR FPs have been designed through mutagenesis, such as eqFP670 [3] and TagRFP675 [4], reaching maximum emission wavelengths of 670 nm and above. Monomeric NIR variants, rather than other forms, such as dimers or tetramers, are important for labeling interest proteins in vivo in order to avoid the labeled proteins aggregation caused by FP oligomerization characteristics. Currently, there are still lack of such NIR proteins. At the beginning of our study, a bright monomeric autocatalytic FP derived from eqFP578 [5,6] of the sea anemone *Entacmaea quadricolor*, mNeptune [7] (Ex/Em: 600/650 nm) was reported, which inherits the high brightness of its precursor mKate [8]. Meanwhile, it remains monomeric state in solution, and was thus selected as a starting protein to perform directed evolution towards further red-shifted emission.

In this study, we used random mutagenesis and structure-guided rational design to develop a variant with the maximum emission wavelength above 680 nm, which was named mNeptune681 (Ex/Em: 604/681 nm), in reference to its maximum emission wavelength. Following additional optimization, twelve further variants were found to possess a further red shift in emission relative to mNeptune681. Among them, one monomer (Ex/Em: 604/684 nm) was termed mNeptune684, which was significantly improved the ability of resisting photobleaching. All variants were characterized for their physical, and possible mechanisms responsible for the redshift are discussed based on 3D structure modeling analysis.

## Materials and Methods

### Mutagenesis and Plasmid Preparation

The cDNAs of FPs (eqFP670, mNeptune and its derivatives) were cloned into the restriction sites BamH I and Sac I sites of the vector pQE30 (Qiagen, Hilden, Germany) using forward primers and reverse primers respectively (S1 Table). To generate mNeptune variants, we mutated the mNeptune gene in every PCR process using site-directed mutation protocol [9]. Here, degenerative primers were used to introduce random mutations at the neighboring residues sites around the chromophore (S2 Table). The design methods of degenerative primers, mutation libraries construction and screening methods are identical with our previous study [10].

### Protein Expression and Purification

eqFP670, mNeptune, mNeptune681 and its derivatives were expressed in a XL1 Blue strain. Bacterial cultures were grown overnight at 37°C and further incubated at 25°C for 12 h according to a method described previously [11]. Cultures were collected by centrifugation, and cell pellets were resuspended in binding buffer (20 mM Tris-HCl, 500 mM NaCl, 20 mM imidazole, pH 8.0) before cell lysis using sonication. The recombinant proteins were purified using Ni-NTA His-Bind resin (GE Healthcare, Bethesda, USA), followed by a gel filtration step using a Superdex-200 size exclusion column (GE Healthcare, Bethesda, USA). After gel filtration, the purified proteins were characterized by SDS-polyacrylamide electrophoresis. Proteins were stored in PBS until further analysis.

### Gel Filtration Assays

Gel filtration analysis was eqFP670, mNeptune, mNeptune681 and its derivatives (approximate 3–4 mg/ml), using a Superdex 200 10/300 GL column (GE Healthcare, Bethesda, USA).

### Spectroscopic Characterization

Absorption spectra and excitation/emission spectra of FPs were measured separately using a LAMBDA-25 UV/Vis spectrophotometer (Perkin Elmer, Waltham, USA) and a LS55 fluorescence spectrophotometer (Perkin Elmer, Waltham, USA) equipped with a red sensitive photomultiplier tube (Hamamatsu Photonics, Hamamatsu, Japan). Molar extinction coefficients were quantified using a alkali denaturation method [12,13]. In order to measure accurately the quantum yields, according to the description of optical dilution measurement [14], protein samples were first diluted in PBS (pH7.4) with UV absorption value in the range of 0.01–0.05. Fluorescence intensity of the mutant FP was compared with that of mNeptune (a quantum yield of 0.2). In order to facilitate the brightness comparisons between different FP variants, the brightness of all the proteins is converted to the relative brightness, which is relative to the brightness of EGFP (the product of quantum yield and extinction coefficient compared to the brightness of EGFP (53,000M^-1^cm^-1^×0.60) [15]).

### pH Titration Assays

FPs was dissolved in a series of buffers of differing pH values, range from pH 3.0 to pH 11.0. Equal volumes (200 μl) of FP solutions(concentration 20 μM) were analyzed using a Synergy H1 plate reader in Costar UV transparent 96-well plates for the absorption spectra. Those p*K*a values were taken as the pH values of the fluorescent proteins, where the absorption or the deprotonation ratio of the chromophore reached 50% of their maxima.

### Photobleaching Measurements

Aqueous droplets of FPs in PBS solution were mixed with mineral oil. After sufficiently vibrating on a vortex apparatus (Genie 2, Bohemia, USA), microdroplets of FPs were prepared with the size of 5–10 μm, to guarantee all FPs in the same irradiation conditions. Photobleaching experiments were carried out using an Olympus IX81 inverted microscope (Olympus, Tokyo, Japan), which was equipped with a standard 561 nm laser light source and 60×1.4 NA oil immersion lens. The FP microdroplets surrounded by mineral oil were continuously illuminated for 10–15 min by a laser output power of 7.2 mW cm^-2^, and images were collected every 0.5 s. Averaged fluorescence signals of FP microdroplets from three independent experiments were quantified using PerkinElmer Volocity 5.3 software. At time zero, the fluorescence signals were normalized to 100%. Photobleaching half-life time was defined as the time point at which the signal dropped to 50% of its initial value.

### Modeling the structures of mNeptune681 and its derivatives

Based on the crystallography structure of eqFP670 (PDB number: 4EDS), the structures of mNeptune681 and its monomeric derivatives(Q159A, Q159N, Q159P and Q159G) were simulated using the alignment mode of homology modeling of the SWISS-MODEL Workspace [16]. The side chain’s spatial structures of mutated amino acids were determined according to the energy minimization.

## Results

### Two near-infrared derivatives of mNeptune, G41Q and G41Q/C158M were obtained in the preliminary screening

**Fig 1:**
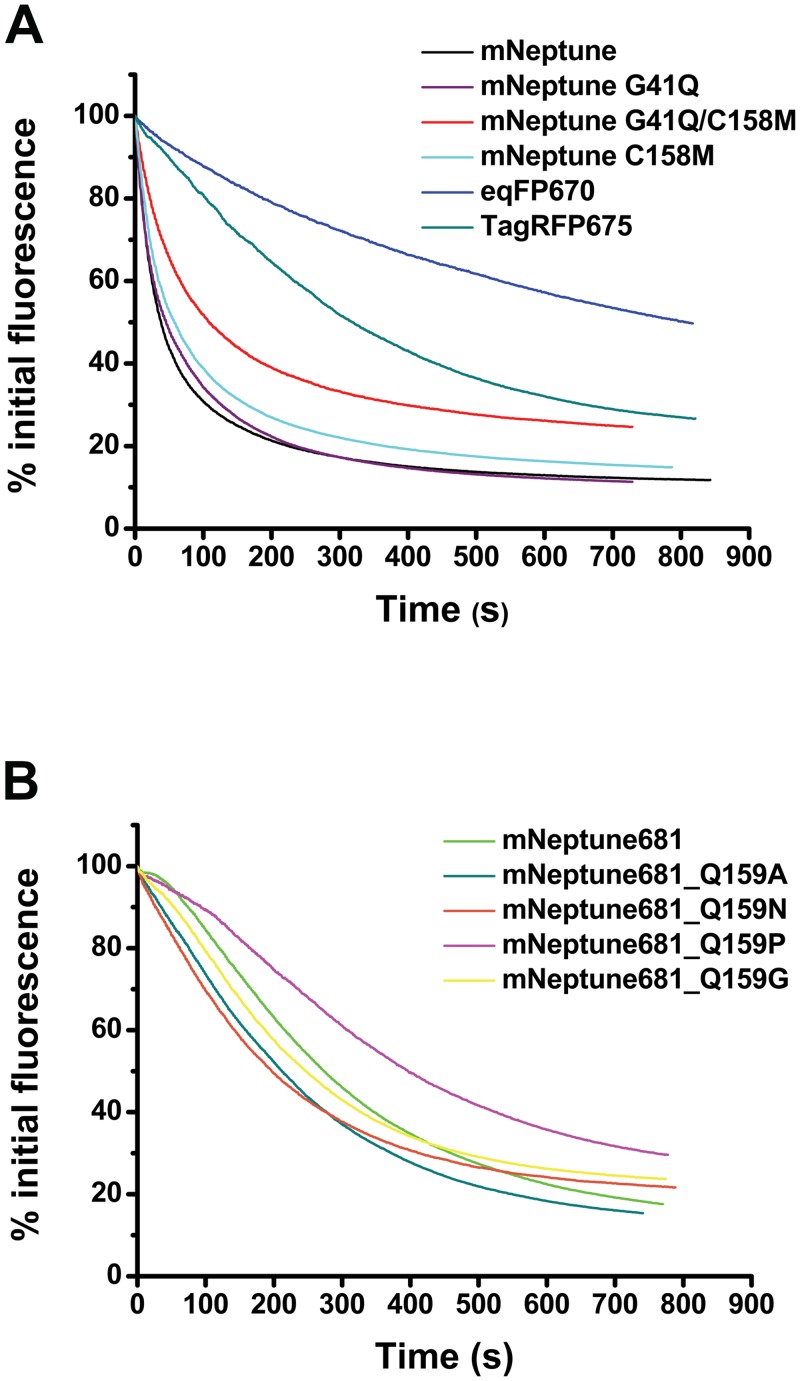
Comparison of photobleaching kinetics. (A) FPs with a maximum emission wavelength of aprox. 670 nm. (B) Monomeric FPs with a maximum emission wavelength above 680 nm.

**Fig 2:**
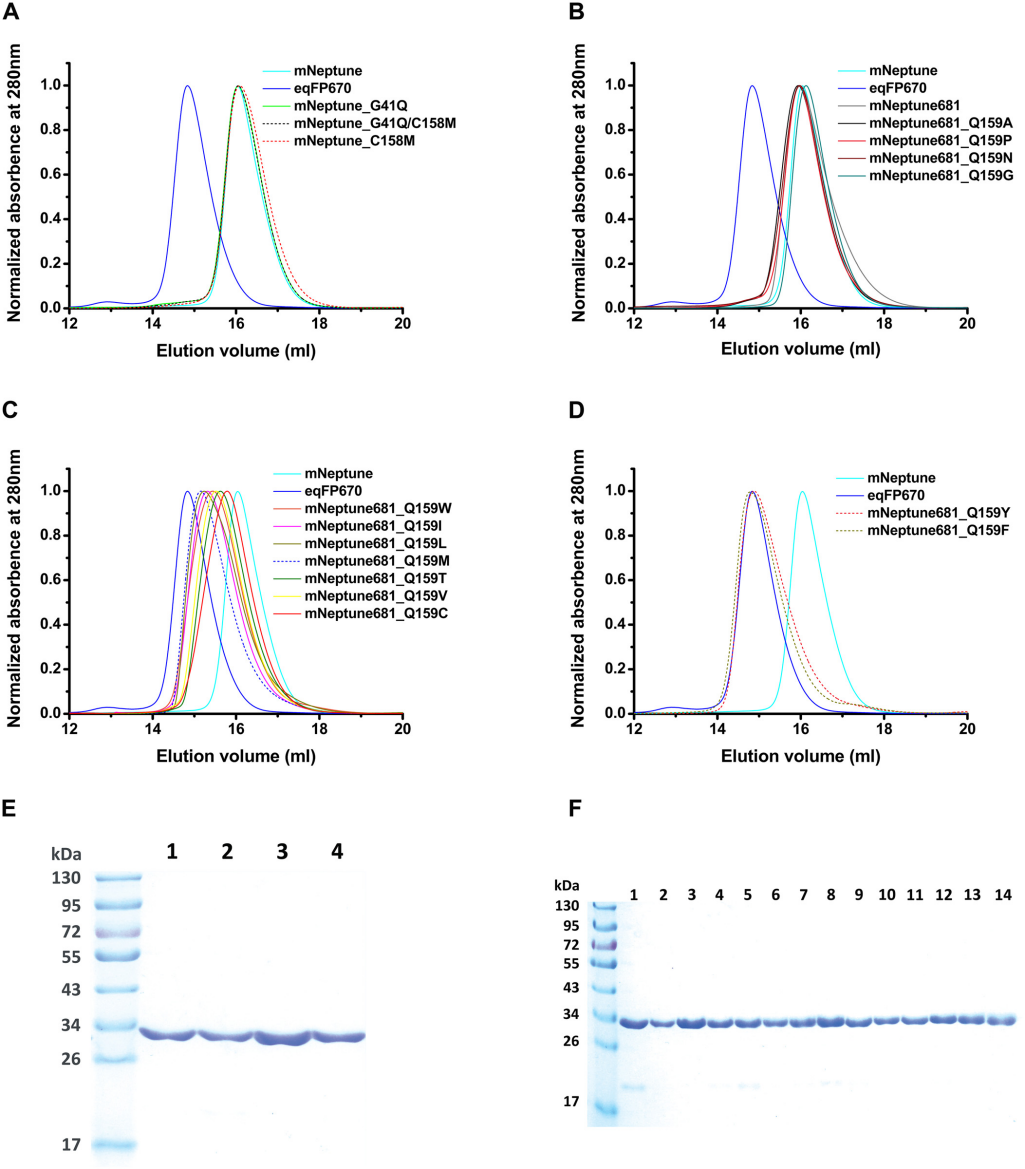
Gel filtration analysis and SDS-PAGE electrophoresis of FPs. Size exclusion chromatography of FPs (approximate 3–4 mg/ml) in PBS using a Supdex 200 10/300GL column. The elution peaks of eqFP670 and mNeptune serve as monomeric and dimeric controls, respectively (see reference 3 and reference 7). The elution volume of eqFP670 (shown as solid blue line) and mNeptune (shown as solid cyan line) were used as the standard reference of dimer and monomer. (A) The variants of mNeptune, G41Q (solid green line), G41Q/C158M (short black dashed line) and C158M (short red dashed line), have the same oligomeric characteristics to that of mNeptune, suggesting that they are monomer. (B) mNeptune681 (solid gray line) and its several monomeric deriveatives, Q159A (solid black line), Q159P (solid pink line), Q159N (solid wine line) and Q159G (solid dark cyan line), with the maximum emission wavelength above 680 nm display the same monomeric property as that of mNeptune. (C) The variants of mNeptune681, Q159W (solid orange line), Q159I (solid magenta line), Q159L (solid dark yellow line), Q159M (short navy dashed line), Q159T (solid olive line), Q159V (solid yellow line) and Q159C (solid red line), with the maximum emission wavelength around 680 nm, display the weak dimeric characteristics. (D) Two independent mutations Q159Y (short pink dashed line) and Q159F (short dark yellow dashed line) in mNeptune681 cause proteins dimerization. (E)-(F) After gel filtration, SDS-Polyacrylamide electrophoresis of FP variants. (E) mNeptune (lane 1), mNeptune_G41Q (lane 2), mNeptune_C158M (lane 3), mNeptune_G41Q/C158M (lane 4). (F) mNeptune681 (lane 1) and its derivatives (lane 2 to 14), they are mNeptune681_Q159G (lane 2), mNeptune_Q159P (lane 3), mNeptune_Q159F (lane 4), mNeptune_Q159Y (lane 5), mNeptune_Q159C (lane 6), mNeptune_Q159V (lane 7), mNeptune_Q159T (lane 8), mNeptune_Q159I (lane 9), mNeptune_Q159L (lane 10), mNeptune_Q159A (lane 11), mNeptune_Q159M (lane 12), mNeptune_Q159N (lane 13), mNeptune_Q159W (lane 14).

In order to screen for red-shifted proteins, we started with mutagenesis using random mutation of the entire protein. Initially, we found two red-shifted mNeptune variants, G41Q and G41Q/C158M, The variant mNeptune_G41Q exhibited an excitation maximum of 589 nm and an emission maximum of 669 nm (Table 1). Compared to its precursor mNeptune, this mutant exhibited an 11 nm blue shift in excitation and a 19 nm red shift in emission, other characterisitics shown in Table 1. Although its brightness decreases by about 40% compared to mNeptune, it exhibits a slightly longer photobleaching half-life time of 45 s, compared with 38 s for mNeptune and a little higher pH stability with a p*K*a of 5.2, compared with a p*K*a of 5.4 for mNeptune (Table 1 and Fig 1A). Importantly, gel filtration analysis showed that mNeptune_G41Q maintained its monomeric state, similar to its precursor (Fig 2A).

**Table 1:** Characteristics of the near-infrared fluorescent protein variants obtained in this study.

Mutants	*E*_x_/*E*_m_ (nm) ^a^	*E*_mol_ (M^-1^cm^-1^) ^b^	QY ^c^	*B*_EGFP_ % ^d^	*pK*a	Oligomeric State	Photobleaching half-life time (S)
mNeptune	600/655	68,000	0.20	42.8	5.4	Monomer	38
	600/650 ^e^	67,000 ^e^	0.20 ^e^	42.1 ^e^	5.4 ^e^	Monomer ^e^	160 ^e^
mNeptune_G41Q	589/669	47,000	0.17	25.1	5.2	Monomer	45
mNeptune_G41Q/C158M	590/674	44,000	0.10	13.8	4.9	Monomer	106
**mNeptune681**	604/681	38,000	0.04	4.8	7.3	Monomer	274
mNeptune681_Q159A	607/684	38,000	0.03	3.6	7.2	Monomer	212
mNeptune681_Q159N	607/685	43,000	0.03	4.1	6.6	Monomer	196
mNeptune681_Q159G	605/685	48,000	0.04	6.0	7.0	Monomer	247
mNeptune681_Q159P(**mNeptune684**)	604/684	39,000	0.03	3.7	6.5	Monomer	396
mNeptune681_Q159C	606/686	44,000	0.02	2.8	7.0	Weak dimer	265
mNeptune681_Q159W	606/685	71,000	0.03	6.7	6.0	Weak dimer	409
eqFP670	603/670	68,000	0.04	8.6	4.5	Dimer	804
	605/670 ^f^	70,000 ^f^	0.03 ^f^	6.6 ^f^	4.5 ^f^	Dimer ^f^	>700 ^f^
TagRFP675	590/674	39,000	0.06	7.4	5.6	Monomer	317
	598/675 ^g^	46,000 ^g^	0.08 ^g^	11.6 ^g^	5.7 ^g^	Monomer ^g^	35 ^g^

All data presented here were from the current experiments with exception of ^e, f^ and ^g^.

^a^ excitation and emission maxima.

^b^ molar extinction coefficient measured by alkali denaturation method (Ref. 12, Ref.^13^)

^c^ quantum yield

^d^ brightness relative to the brightness of EGFP [the product of quantum yield and extinction coefficient compared to the brightness of EGFP (53,000M^-1^cm^-1^×0.60)].

^e^ Data from reference 7 as the control.

^f^ Data from reference 3 as the control.

^g^ Data from reference 4 as the control.

mNeptune_G41Q/C158M displayed amaximum emission wavelength of 670 nm (Ex/Em: 590/674 nm), and exhibited a molar extinction coefficient of 44,000 M^-1^cm^-1^ and quantum yield of 0.1 (Table 1). Although its brightness further decreased relative to mNeptune_G41Q, two remarkable characteristics were observed, namely its improved pH-stability and photostability, with a p*K*a of 4.9 and a photobleaching half-life time of 106 s, compared to the two other forms mNeptune and G41Q (Table 1 and Fig 1A). Gel filtration analysis showed that the evolution volume of mNeptune_G41Q/C158M is nearly the same with that of mNeptune, indicating that mNeptune_G41Q/C158M also maintains its monomeric state in solution similar to mNeptune (Fig 2A). TagRFP675 is the only monomer with a maximum emission wavelength breaking 670 nm prior to our study. According to our characterization on mNeptune_G41Q/C158M and TagRFP675, we found that mNeptune_G41Q/C158M has a similar molar extinction coefficient, quantum yield and brightness with that of TagRFP675, while has shorter photobleaching half-life time (106 s versus 317 s) and higher pH-stability (p*K*a of 4.9 versus p*K*a of 5.7) (Table 1 and Fig 1A).

### Creating an extensive hydrogen-bond network in mNeptune_G41Q to obtain a new NIR protein mNeptune681

**Fig 3:**
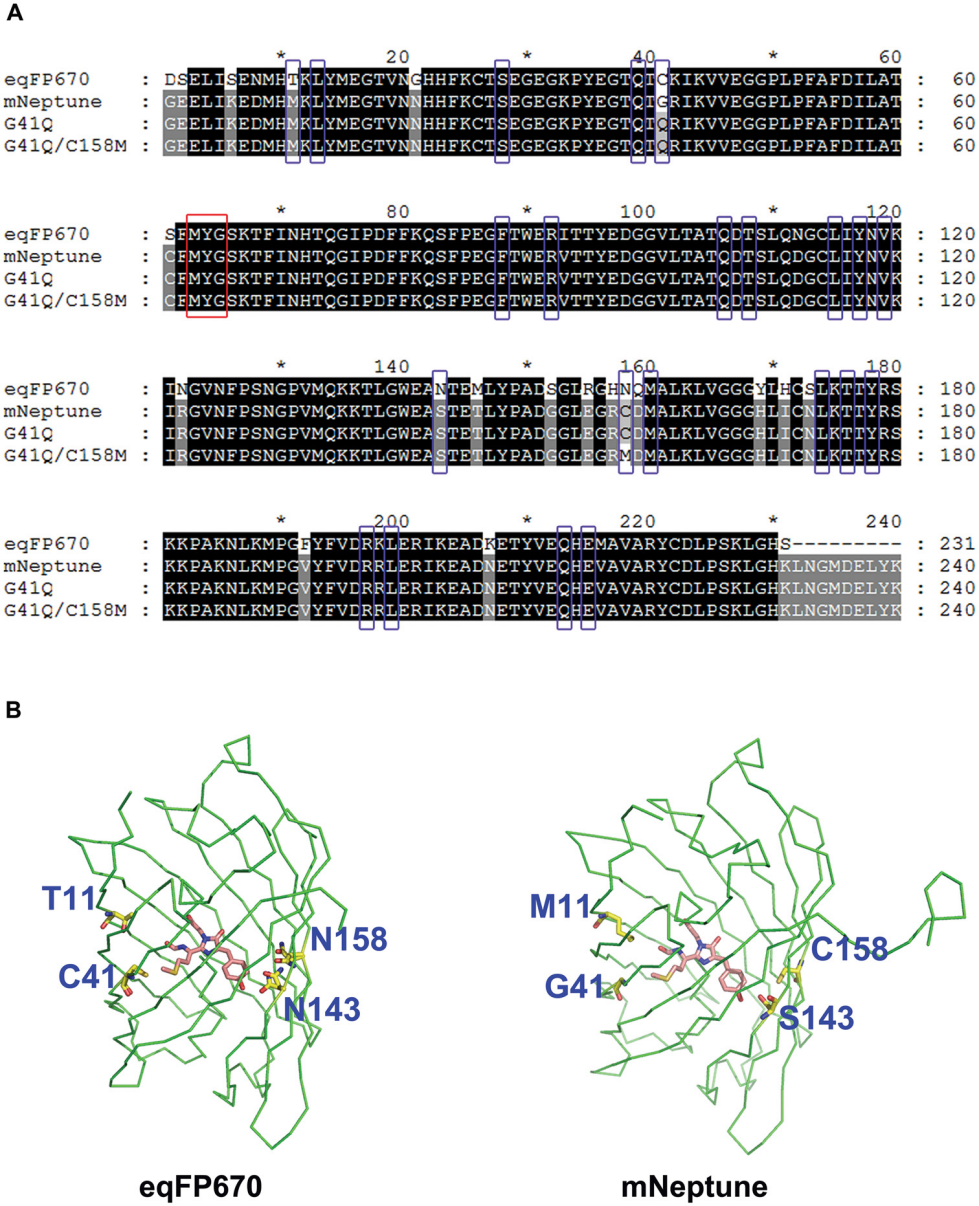
Sequence alignments and structures comparison of mNeptune’s two NIR mutant forms with NIR FP eqFP670. (A) Amino-acids in the vicinity of chromophore are marked with blue rectangles, the chromophore is marked with a red rectangle. (B) The 3D structures of eqFP670 and mNeptune are marked with green ribbon using PyMol software, and the chromophore (pink color) and its surrounding different amino acids (yellow color) between two proteins are labeled with Sticks pattern. amino-acid differences adjacent to the chromophore are marked in blue showed in the crystal structure diagrams.

A previous study [17] showed that NIR FP eqFP670, a homologous protein of mNeptune, possesses an extensive hydrogen-bond network that is mainly constructed by Asn143 and Asn158, resulting in a large redshift in emission relative to its precursor Katushka [18]. We combined this insight with 3D structure-based rational design to develop mutants with an even larger redshift NIR. Sequence and structure alignments were performed for our two mutant forms with NIR FP eqFP670 (Fig 3). Our results showed that almost all amino acids with side chains oriented towards the chromophore are identical, with the exception of the residues 11, 41, 143 and 158 (Fig 3A). Furthermore, 3D structure analysis of mNeptune against eqFP670 showed that residues 11, 41 and residues 143, 158 locate at the two opposite ends of the π-conjugated plane of the chromophore (Fig 3B). Thus, we first introduced two mutations S143N and C158N into mNeptune_G41Q. To our surprise, a blue shift was observed, rather than a red shift in this new variant G41Q/S143N/C158N form. As a result, it exhibited an excitation maximum of 452 nm and emission maximum of 541 nm (Table 2), which indicates that the two asparagine are not enough to form the extensive hydrogen-bond network, and related mechanisms may be more complicated. Meanwhile, the same blue shift in emission was observed when the two asparagine were introduced into mNeptune (Table 2).

**Table 2:** Contributions of individual mutations to red-shifting in mNeptune681.

Mutants	*E*_x_/*E*_m_ (nm) ^a^	*E*_mol_ (M^-1^cm^-1^) ^b^	QY ^c^	*B*_EGFP_ % ^d^
mNeptune_G41Q/S143N/C158N/E155R/R157H/D159Q(**mNeptune681**)	604/681	38,000	0.04	4.8
mNeptune681_R155E	604/677	30,000	0.02	1.9
mNeptune681_H157R	606/667	58,000	0.01	1.8
mNeptune681_Q159D	450/541	20,000	0.23	8.7
mNeptune681_R155E/H157R	607/671	37,000	0.03	3.5
mNeptune681_R155E/Q159D	451/545	31,000	0.31	30.2
mNeptune681_H157R/Q159D	453/540	22,000	0.21	14.5
mNeptune_G41Q/S143N/C158N	452/541	29,000	0.13	11.9
mNeptune_S143N/C158N	452/545	16,000	0.11	5.5

^a^ excitation and emission maxima.

^b^ molar extinction coefficient measured by alkali denaturation method (Ref.^13^)

^c^ quantum yield

^d^ brightness relative to the brightness of EGFP

**Fig 4:**
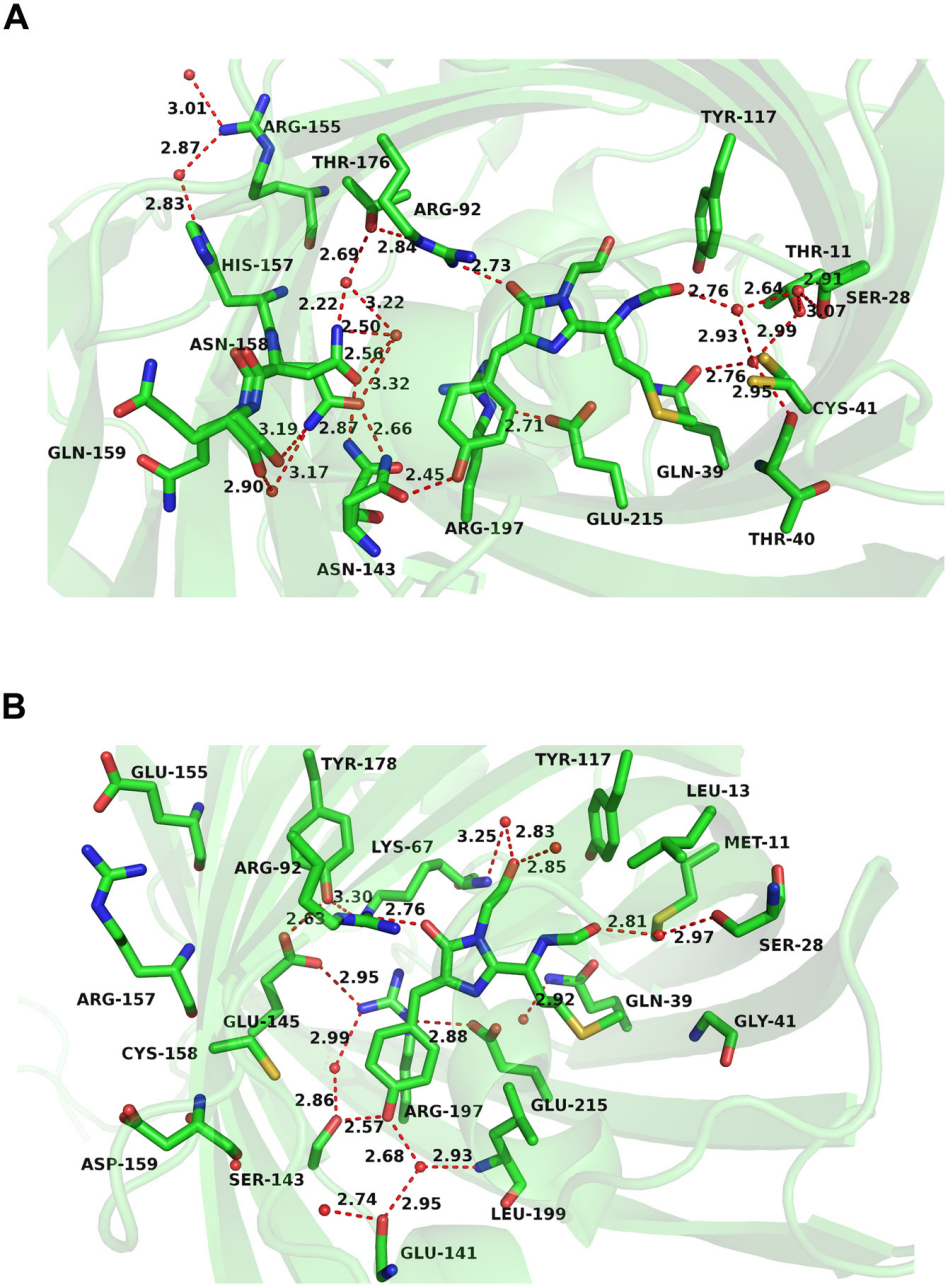
Immediate chromophore hydrogen bonds environment of (A) eqFP670 (PDB entry: 4EDS), (B) mNeptune (PDB entry: 3IP2). Water molecules and hydrogen bonds measured in Å are shown as red spheres and dashed red lines, respectively.

**Fig 5:**
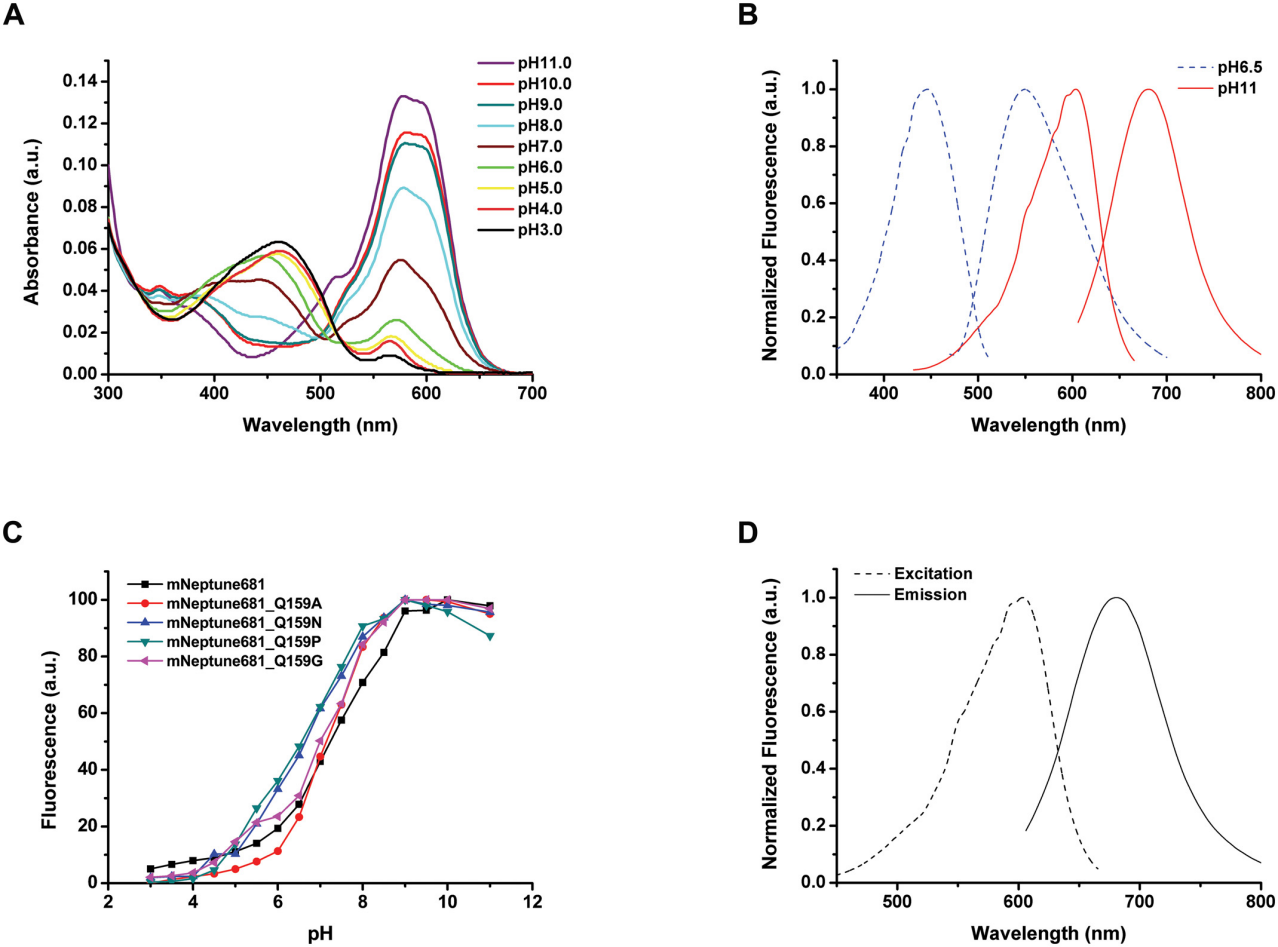
Spectral properties of mNeptune681. (A) Absorbance spectra at pH 3.0–11.0. (B) Fluorescence excitation (dashed line) and emission (solid line) spectra at pH 6.5 (blue line) and pH 11.0 (red line). (C) Equilibrium pH dependence of fluorescence emission intensity for mNeptune681 and its monomeric variants. mNeptune681 (black line), mNeptune681_Q159A (red line), mNeptune681_Q159N (green line), mNeptune681_Q159P (blue line), mNeptune681_Q159G (Cyan line). (D) Fluorescence excitation (dashed line) and emission (solid line) spectra of mNeptune681.

After analyzing the primary sequences and X-ray crystal structures of eqFP670 and mNeptune, we found that they also differ in three residue, namely 155, 157, 159, apart from the two residues at positions 143 and 158 between eqFP670 and mNeptune_G41Q (Fig 3A), and all the side chains of the three residues locate at the external of the β-barrel (Fig 4). The three amino acids respectively are Arg155, His157 and Gln159 in eqFP670 (Fig 4A), while are Glu155, Arg157 and Asp159 in mNeptune (Fig 4B). Based on these results, we speculated that Arg155, His157 and Gln159 may be also involved in the construction of extensive hydrogen-bond network through the way of hydrogen-bonding interaction with two asparagine. To test our surmise, we introduced three mutations E155R, R157H and D159Q into mNeptune_G41Q/S143N/C158N. The results of spectrum scanning showed that the final variant mNeptune_G41Q/S143N/C158N/E155R/R157H/D159Q produced a similar NIR fluorescence emission caused by the single mutant form G41Q, and displayed a 12 nm of red shift in emission relative to its precursor mNeptune_G41Q (Table 1). Taken together, this suggest that Arg155, His157 and Gln159 indeed participate in the construction of the extensive hydrogen-bond network, supporting our assumption. At last, in accordance with the current nomenclature convention, we named this variant G41Q/S143N/C158N/E155R/R157H/D159Q as mNeptune681 (Ex/Em: 604/681 nm) (Table 1, Fig 5D).

### Contributions of individual mutations to the red-shift in mNeptune681

Since that three additional substitutions (E115R, R157H and D159Q) restored the NIR fluorescence and generated further redshift, six different combinations of reverse mutations at the three positions were performed to investigate the individual roles of these amino acids (Table 2). Our results showed that only those mutants that possess a Glutamine at position 159 could emit a fluorescence signal at around 670 nm, similar to their precursor mNeptune_G41Q, indicating that Gln159 is essential for maintaining NIR fluorescence. Furthermore, we also found that successive incorporation of His157 and Arg155 into the D159Q mutant result in 6 nm and 4 nm redshift in emission, respectively (Table 2). Thus, the individual contributions of the three amino acids in mNeptune681 to the redshift were identified.

### Saturation mutagenesis of residue position 159 in mNeptune681 yielded additional redshifts in emission

**Fig 6:**
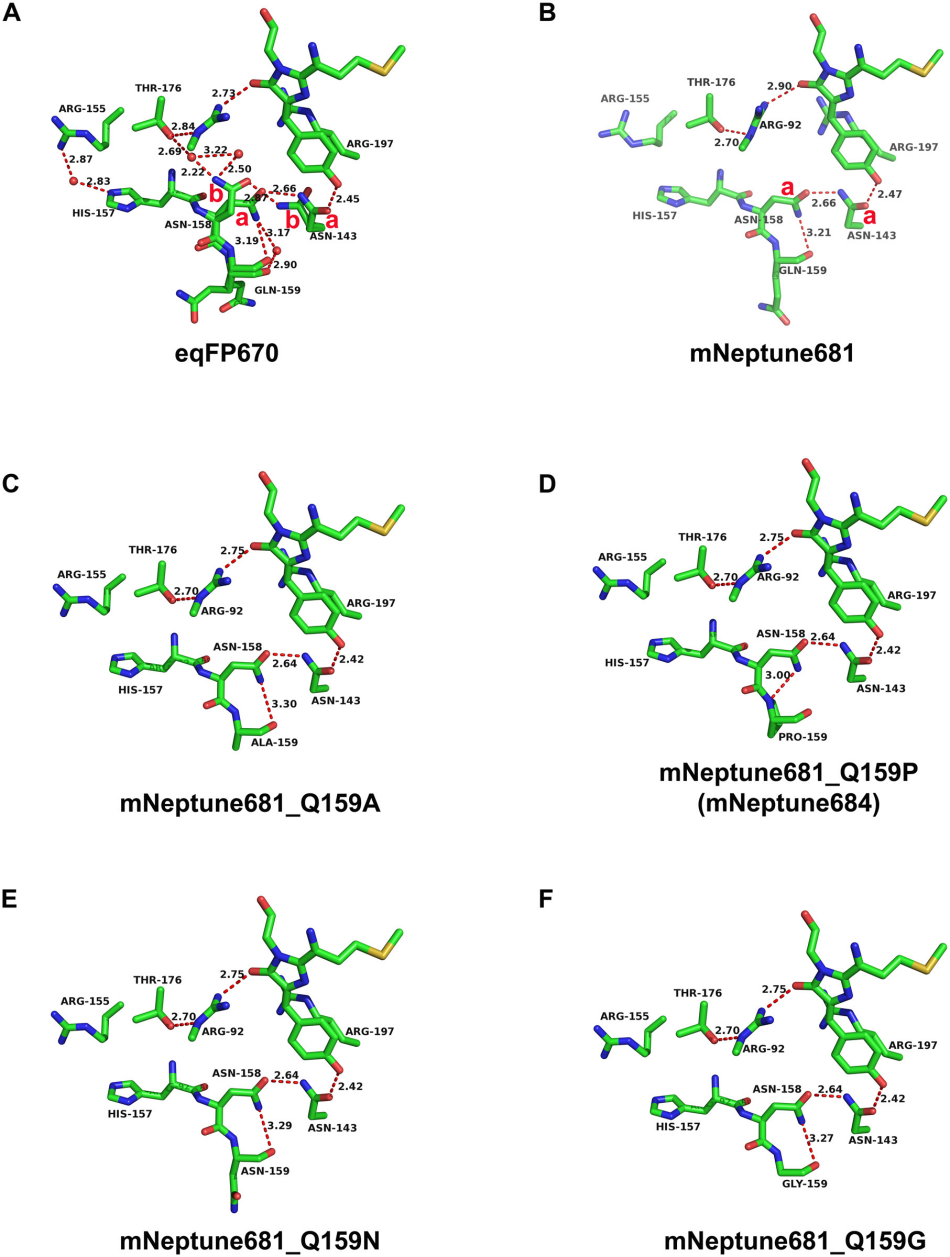
The extensive hydrogen-bond network resulting in the NIR emission of eqFP670 and simulated structures of mNeptune681 and its monomeric derivatives. a and b in red color in the diagram stand for the majority conformation state and the minority conformation respectively. (A) From the crystal structure of eqFP670 (PDB entry: 4EDS). (B)-(F) From the simulated structures of mNeptune681 and its monomeric derivatives with the residue 159 mutations using homology modeling method based on the crystal structure of eqFP670. Immediate chromophore hydrogen-bond environment of (A) eqFP670, (B) mNeptune681, (C) mNeptune681_Q159A, (D) mNeptune684, (E) mNeptune681_Q159N and (F) mNeptune681_Q159G. Water molecules and hydrogen bonds measured in Å are shown as red spheres and red dashed lines, respectively. The hydrogen-bonds between residue 159 and Asn158 of these mutants are crucial to maintaining NIR fluorescence emission. Among these hydrogen-bonds, a distinctive hydrogen-bond was found in the variant Q159P, which is located between the alpha-amino nitrogen atom of Pro159 and the amide nitrogen atom of Asn158, and is the strongest and most stable hydrogen-bond due to its shortest length of 3.00 Å.

Given the importance of the Gln159 to the redshift, analysis on the hydrogen-bond environments of Gln159 and the chromophore surroundings indicated that an additional hydrogen-bond exists between the carboxyl oxygen of Gln159 and the amide nitrogen of Asn158 (Fig 6B), and it has the length of 3.21 Å which is shorter than 3.19 Å of eqFP670, suggesting that it is a weak hydrogen-bond. In order to strengthen the hydrogen-bond interaction between Asn158 and residue 159, saturation mutagenesis was performed at position 159. As a result, a dozen of other variants were also found with an NIR emission above 680 nm. Of these variants, four mutant forms (Q159A, Q159N, Q159P and Q159G) displayed a slightly redshift in emission relative to mNeptune_681, and their maximum emission wavelengths range from 681 to 685 nm (Table 1).

### Characterization of the near-infrared variant mNeptune681 and its derivatives

We first characterized the biochemical and physical properties of mNeptune681. Gel filtration analysis showed that the elution volume of mNeptune681 is similar to that of mNeptune in PBS buffer (protein concentration 3–4 mg/ml, pH7.4), suggesting that mNeptune681 maintains the monomeric state in solution (Fig 2B). The purified protein displayed a molar extinction coefficient of 38,000 M^-1^cm^-1^, with a quantum yield of 0.04, which results in a lower brightness relative to mNeptune_G41Q. In addition, its pH-stability also decreased visibly, with a p*K*a of 7.3 (Table 1), compared with 5.4 of mNeptune. The results of spectrum scanning showed that mNeptune681 exhibits a strong dependence of emission maximum on the excitation wavelength and pH of buffer similar to other NIR FPs. As shown in Fig 5A–5C, at pH < 7.0, mNeptune681 gradually exhibited strong absorption centered at 460 nm, corresponding to the neutral DsRed-like chromophore. Excitation at 460 nm yielded strong fluorescence with an emission peak at 550 nm. A minor peak of absorption was found at 570 nm, which corresponds to a weak emission of around 637 nm. In contrast, at pH > 7.0, the absorbance spectrafeatured a major band with a maxima at around 580 nm that corresponded to the anionic DsRed-like chromophore.

Next, we characterized mutants derived from saturation mutations at residue position 159 of mNeptune681. Gel filtration analysis demonstrated that four mutants of mNeptune681, namely Q159P, Q159N, Q159A and Q159G remained in the same monomeric state as mNeptune681 (Table 1, Fig 2B). The other properties of all derivatives are listed in Table 1, and shown in Fig 1B and Fig 2B–2D. From these data, three most prominent features about these variants can be summarized. Firstly, mNeptune681_Q159C has the longest emission wavelength (emission maxima at 686 nm) among all FPs reported so far. Secondly, mNeptune681_Q159W exhibits the highest brightness, which is equivalent to that of eqFP670 and about 6.7% of that of EGFP. Thirdly, among the five monomeric variants that possess an emission maximum above 680 nm, mNeptune681_Q159P displays the best performance, namely highest pH stability with a p*K*a of 6.5, an emission peak of 684 nm and the longest photobleaching half-life time (Table 1, Fig 1B). Therefore, it was named mNeptune684.

### Comparison of the strength of hydrogen bonds between different residue 159 variants

In order to compare the strength of hydrogen-bonds between different residue 159 variants, we simulated the structures of these variants using homology modeling method based on the crystallographic structure of eqFP670. As shown in Fig 6B–6F, the length of the hydrogen-bond between residue 159 and Asn158 ranges from 3.00 Å to 3.30 Å. This difference in distance probably results in different strengths of interaction between Asn158 and the various amino acid residues at position 159. Variant Q159A displays the longest hydrogen-bond length with Asn158, while Q159P has the shortest. This may explain why Q159P has more excellent optical performance.

## Discussion

It is generally accepted that the diversity of emission maxima in FPs results from a combination of two factors: chemical modifications to the chromophore structures, and interactions occurring between the chromophore (both in the ground and excited states) and its immediate environment. mNeptune used in this study has been reported earlier to possess a water-mediated hydrogen bond between the acylimine oxygen of the chromophore and the neighboring residue Ser28 (Ref. 7), and in other red-shifted FPs such as mPlum [19,20] also exists the hydrogen-bonds between the chromphore and its immediate protein matrix. Just these hydrogen-bond interactions finally leads to these proteins further red-shifted emission relative to their precursor proteins. In short, the more hydrogen-bond donations occur around the chromophore, the more electron density is accumulated around the chromophore, and the more red-shifted emission bands are.

In the beginning of this study, we firstly screened out two red-shifted mutants, mNeptune_G41Q and mNeptune_G41Q/C158M. Among them, G41Q mutation is the immediate reason for the significant redshift in emission. Just for this amino-acid substitution in mNeptune, a shorter direct hydrogen-bond forms between the amide nitrogen atom of Gln41 and the acylimine oxygen of chromophore, suggesting that the hydrogen-bonding interaction between them is enhanced. As a result of this strengthened hydrogen-bonding interaction between the chromophore and protein matrix, the chromophore conjugated π–electron system in the excited state become more stable, finally causing the redshift in emission. The detailed redshift mechanism had been reported by Verkhusha (Ref. 4).

In order to evolve the more red-shifted FPs, we then attempted to introduce a hydrogen-bond network similar to that of eqFP670 around the chromophore in the variant mNeptune_G41Q. After some optimizations, a series of redshifts beyond 680 nm, e.g. mNeptune681, mNeptune684 etc. were finally obtained (Table 1), which exhibit the largest Stokes shift (up to 80 nm) compare to the existing conventional FPs. 3D structure analysis on the crystal structure of eqFP670 revealed that both Asn143 and Asn158 locate at the side of the chromophore *p*-hydroxyphenyl ring and lie in approximately the same plane, and both the side chains’ conformation of the two asparagine are able to change freely and form two alterable states that respectively in crystals (Fig 6A). One is the major conformation state, and the other is minor conformation state. As in the Fig 6A and 6B, a and b stand for the majority conformation state and the minority conformation respectively (Ref. 17). Seeing that the side chains of the two asparagine can occur conversions between major and minor conformation, thus we can conclude that the hydrogen bonds between the two asparagine and the hydrogen-bond connection between Asn143 and the chromophore are both not very stable.

In order to stabilize these hydrogen bonds, we carefully analyzed the amino-acids surrounding the chromophore and two asparagine, and made some mutagenesis. Interestingly, apart from two asparagine involved in the construction of the hydrogen-bond network, three other important amino acids (Arg155, His157, Gln159) are also found to be crucial to the stability of this hydrogen-bond network. Jointing with reverse mutagenesis results (Table 2) and our analysis on 3D structures of proteins, we believed that successive introductions of three amino acids should help immobilizing the spatial conformation of the side chains of Asn158, and allows Asn158 to adopt the major conformation as much as possible (Fig 6A and 6B). Specifically speaking, the hydrogen bond between residue Gln159 and Asn158 can partially fix the position of the side chains of Asn158 near the side of Gln159, on the other hand, the overall spatial conformation of His157 was immobilized by the hydrogen-bond interaction between itself and Arg155, thereby its main chain carbonyl oxygen atom forms a steric effect to the minor conformation state of Asn158’s side chain (Fig 6A and 6B). When Asn143 is present in the major conformation state towards the chromophore, its side-chain amide group is able to form two strong hydrogen bonds: one is an unusually short hydrogen bond with the hydroxyl oxygen of the p-hydroxyphenyl group of the chromophore, and the other is a hydrogen bond with the oxygen atom of the side chain of Asn158 in its major conformation (Fig 6A and 6B). Therefore, through these hydrogen-bond connections, the hydrogen-bond network around the chromophore is further extended. As a result, the chromophore acquires more hydrogen-bond donations from the surrounding protein matrix, and accumulates more electron densities, finally causing a significant redshift in emission of mNeptune681 relative to mNeptune_G41Q.

In addition to a longer emission wavelength, other characteristics are also important for successful application of NIR FPs, such as monomeric properties, higher brightness, faster maturation time, higher photostability and higher pH-stability. In the current study, mNeptune684 (mNeptune681_Q159P) exhibits a highly improved photostability thanks to Pro159. This is possibly supported by structural simulation of mNeptune684. The result of homology modeling indicates that a distinctive hydrogen-bond exists between the alpha-amino nitrogen atom of Pro159 and the amide nitrogen atom of Asn158 (Fig 6D), which the length is 3.00 Å shorter than that observed in mNeptune681 (3.21 Å) (Fig 6B). This illustrates that the hydrogen-bonding interaction of mNeptune684 may be stronger and more stable. In view of the fact that the simulated side chain’s spatial structures are only the preliminary speculations, thus they still need to be further confirmed by the approach of quantum mechanics/molecular mechanics (QM/MM), and the detailed mechanisms still need to be thoroughly analyzed. Moreover, the cyclic side chain of Pro itself is a rigid structure. Consequently, the introduction of Pro159 turns the spatial position of the side chain of Asn158 into a more fixed point, possibly causing the entire hydrogen-bond network to become more rigid. Under this more rigid environment, chromophore is packed more closely, thus its mobility may be reduced. When the chromophore is irradiated by the excitation light, perhaps the decay of emission light energy caused by chromophore mobility slow down, thereby corresponding to the enhancement of photostability. This phenomenon of improvement in photostability caused by a decreased chromophore mobility was also observed in a blue fluorescent protein Azurite [21], which exhibits a 40-fold increase in photobleaching half-life over its precursor wild type BFP.

Based on the above analysis, we learn that two different types of hydrogen-bond interactions around the chromophore are respectively responsible for the extended Stokes shifts and the more red-shifted emission, which finally generate our NIR variants with the largest Stokes shifts (about 80 nm) among known GFP-like proteins. The expanded Stokes shifts helps to decrease mutual interference between excitation and emission lights during imaging and thus reduces the background noise. However, the expanded Stokes shifts might also lower the quantum yields and brightness. When using these near infrared FPs developed by us for tissue imaging, brightness also needs to be taken into account.

Throughout the application history of fluorescent proteins, due to their intrinsic high brightness and multiple colors, some FPs, such as GFP, RFP and far-red FPs, have been used for whole-body imaging of tumors [22] and *in vivo* imaging with subcellular resolution [23]. These proteins have allowed people to visualize, in real time, important aspects of cancer in living animals, including tumor cell mobility, invasion, metastasis and angiogenesis [24, 25]. The near-infrared variants developed in our study has two advantages apparently. First, longer excitation wavelength (above 600 nm) with its lower energy enables real-time imaging to take place without harming the animals’ cells and tissues. Second, emission wavelengths above 680 nm is in the “optical window”, where light has its maximum depth of penetration in tissue [26]. Even so, the brightness of our near-infrared FP variants has been weakened along with the redshift in emission. Comprehensive assessment of these variants for *in vivo* imaging application is expected in next step.

In conclusion, through directed evolution, we obtained a number of valuable NIR FP variants which exhibit significant red shift in emission than the existing NIR FPs. These mutants provide a NIR FP toolbox which help labeling a variety of molecule events in living organisms. Our success in red-shifting the emission maxima from 650 nm to 685 nm indicates that there is still space for improving fluorescent proteins with desired properties.

## Supporting Information

S1 Table The primers for construction of FP clones. (DOCX)

S2 Table The selected sites by rational design and the primers used for site-directed mutations. (DOCX)
