# Supplementary material for: Mutagenesis of mNeptune Red-Shifts Emission Spectrum to 681-685 nm
Source: PLoS One. 2016 Apr 27;11(4):e0148749. doi: 10.1371/journal.pone.0148749 (PMC4847776; doi:10.1371/journal.pone.0148749)
Supplement: S1 Table — (DOCX) [file pone.0148749.s001.docx]

**S1 Table.** The primers for construction of FP clones.

| **Fluorescent proteins** | **Forward primers** | **Reverse primers** |
| --- | --- | --- |
| mNeptune and its derivatives | 5' ATAGGATCCATGGTGTCTAAGGGCG  AAGAGCTGATTA 3' | 5' ATAGAGCTCTTACTTGTACAGCTCGT  CCATGCCATTA 3' |
| eqFP670  TagRFP675 | 5' ATATGGATCCATGGGAGAGGATAGC  GAGCTGATCTCCGAG 3'  5' ATATGGATCCATGAGTGAGCTGATTA  AGGAGAACATGCACATGAAGCTG 3' | 5' TATAGAGCTCTAGCTGTGCCCCAGTT  TGCTAGGCAGGTCG 3'  5' GCATGAGCTCTTAATTAAGTTTGTGC  CCCAGTTTGCTAGGGAG 3' |
