# Supplementary material for: Mutagenesis of mNeptune Red-Shifts Emission Spectrum to 681-685 nm
Source: PLoS One. 2016 Apr 27;11(4):e0148749. doi: 10.1371/journal.pone.0148749 (PMC4847776; doi:10.1371/journal.pone.0148749)
Supplement: S2 Table — (DOCX) [file pone.0148749.s002.docx]

**S2 Table.** The selected sites by rational design and the primers used for site-directed mutations.

| **Mutation sites** | **Degenerative primers** |
| --- | --- |
| Met11 | 5' TTAAGGAGAACATGCAC**NNN**AAGCTGTACATGGAGGG 3'  5' CCCTCCATGTACAGCTT**NNN**GTGCATGTTCTCCTTA 3' |
| Leu13 | 5' GAACATGCACATGAAG**NNN**TACATGGAGGGCACCGT 3'  5' ACGGTGCCCTCCATGTA**NNN**CTTCATGTGCATGTT 3' |
| Met11, Leu13 | 5' ATTAAGGAGAACATGCAC**NNN**AAG**NNN**TACATGGAGGGCACCGT 3'  5' ACGGTGCCCTCCATGTA**NNN**CTT**NNN**GTGCATGTTCTCCTTA 3' |
| Ile70 | 5' CGGCAGCAAGACCTTC**NNN**AACCACACCCAGGGCATCC 3'  5' GGATGCCCTGGGTGTGGTT**NNN**GAAGGTCTTGCTGCCGTA 3' |
| Trp140 | 5' GAAGAAAACACTCGGC**NNN**GAGGCCTCCACCGAGACG 3'  5' CGTCTCGGTGGAGGCCTC**NNN**GCCGAGTGTTTTCTTC 3' |
| Ser143 | 5' ACTCGGCTGGGAGGCC**NNN**ACCGAGACGCTGTACC 3'  5' GGTACAGCGTCTCGGT**NNN**GGCCTCCCAGCCGAGT 3' |
| Cys158 | 5' GCGGCCTGGAAGGCAGA**NNN**GACATGGCCCTGAAGCTC 3'  5' GAGCTTCAGGGCCATGTC**NNN**TCTGCCTTCCAGGCCGCCGT 3' |
| Met160 | 5' GGAAGGCAGATGCGAC**NNN**GCCCTGAAGCTCGTGG 3'  5' CCACGAGCTTCAGGGC**NNN**GTCGCATCTGCCTTCCAGGC 3' |
| Cys172 | 5' CGGGGGCCACCTGATC**NNN**AACCTGAAGACCACATACA 3'  5' ATGTGGTCTTCAGGTT**NNN**GATCAGGTGGCCCCCGCC 3' |
| Leu174 | 5' CCACCTGATCTGCAAC**NNN**AAGACCACATACAGA 3'  5' TCTGTATGTGGTCTT**NNN**GTTGCAGATCAGGTG 3' |
| Val195 | 5' CCCGGCGTCTACTTT**NNN**GACAGAAGACTGGAAAGA 3'  5' TCTTTCCAGTCTTCTGTC**NNN**AAAGTAGACGCCG 3' |
| Arg197 | 5' GCGTCTACTTTGTGGAC**NNN**AGACTGGAAAGAATCAAGGA 3'  5' CCTTGATTCTTTCCAGTCT**NNN**GTCCACAAAGTAGACGC 3' |
| Ala217 | 5' CGAGCAGCACGAGGTG**NNN**GTGGCCAGATACTGCGA 3'  5' TCGCAGTATCTGGCCAC**NNN**CACCTCGTGCTGCTC 3' |
| Val195, Arg197 | 5' AGATGCCCGGCGTCTACTTT**NNN**GAC**NNN**AGACTGGAAAGAATCAAGGAGG 3' |
|  | 5' CTCCTTGATTCTTTCCAGTCT**NNN**GTC**NNN**AAAGTAGACGCCGGGCATCTT 3' |
